# Violence in public transportation: an approach based on spatial analysis

**DOI:** 10.11606/S1518-8787.2017051007085

**Published:** 2017-12-04

**Authors:** Daiane Castro Bittencourt de Sousa, Cira Souza Pitombo, Samille Santos Rocha, Ana Rita Salgueiro, Juan Pedro Moreno Delgado

**Affiliations:** 1 Universidade Federal da Bahia. Escola Politécnica. Departamento de Engenharia de Transportes e Geodésia. Salvador, BA, Brasil; II Universidade de São Paulo. Escola de Engenharia de São Carlos. Departamento de Engenharia de Transportes. São Carlos, SP, Brasil; III Universidade Federal do Ceará. Centro de Ciências. Departamento de Geologia. Fortaleza, CE, Brasil

**Keywords:** Theft, statistics & numerical data, Transportation, Spatial Analysis, Geographic Information Systems, utilization, Violence, prevention & control, Roubo, estatística & dados numéricos, Transportes, Análise Espacial, Sistemas de Informação Geográfica, utilização, Violência, prevenção & controle

## Abstract

**OBJECTIVE:**

To carry out a spatial analysis of the occurrence of acts of violence (specifically robberies) in public transportation, identifying the regions of greater incidence, using geostatistics, and possible causes with the aid of a multicriteria analysis in the Geographic Information System.

**METHODS:**

The unit of analysis is the traffic analysis zone of the survey named *Origem-Destino*, carried out in Salvador, state of Bahia, in 2013. The robberies recorded by the Department of Public Security of Bahia in 2013 were located and made compatible with the limits of the traffic analysis zones and, later, associated with the respective centroids. After determining the regions with the highest probability of robbery, we carried out a geographic analysis of the possible causes in the region with the highest robbery potential, considering the factors analyzed using a multicriteria analysis in a Geographic Information System environment.

**RESULTS:**

The execution of the two steps of this study allowed us to identify areas corresponding to the greater probability of occurrence of robberies in public transportation. In addition, the three most vulnerable road sections *(Estrada da Liberdade, Rua Pero Vaz*, and *Avenida General San Martin)* were identified in these areas. In these sections, the factors that most contribute with the potential for robbery in buses are: F1 - proximity to places that facilitate escape, F3 - great movement of persons, and F2 - absence of policing, respectively.

**CONCLUSIONS:**

Indicator Kriging (geostatistical estimation) can be used to construct a spatial probability surface, which can be a useful tool for the implementation of public policies. The multicriteria analysis in the Geographic Information System environment allowed us to understand the spatial factors related to the phenomenon under analysis.

## RESUMO

**OBJETIVO:**

Realizar uma análise espacial da ocorrência de atos de violência (específicamente roubos) em transporte público, identificando as regiões de maior incidência, por meio da geoestatística, e possíveis causas com auxílio de análise multicritério em Sistema de Informação Geográfica.

**MÉTODOS:**

A unidade de análise é a zona de tráfego da pesquisa Origem-Destino, realizada em Salvador, BA, Brasil, em 2013. Os roubos registrados pela Secretaria de Segurança Pública da Bahia, no mesmo ano, foram localizados e compatibilizados aos limites das zonas de tráfego e, posteriormente, associados aos respectivos centroides. Após determinação das regiões de maior probabilidade de ocorrências, foi feita uma análise geográfica de possíveis causas na região de maior potencial, considerando fatores analisados por meio de uma análise multicritério em ambiente de Sistema de Informação Geográfica.

**RESULTADOS:**

A execução das duas etapas deste trabalho permitiu identificar áreas correspondentes à maior probabilidade de ocorrência de roubo em transporte público. Além disso, foram identificados três trechos viários (Estrada da Liberdade, Rua Pero Vaz e Avenida General San Martin) mais vulneráveis, localizados nessas áreas. Nesses trechos, os fatores que mais contribuem para o potencial de ocorrência de roubo em ônibus são: F1 - proximidade a locais que facilitam a fuga, F3 - grande movimentação de pessoas e F2 - ausência de policiamento, respectivamente.

**CONCLUSÕES:**

Com o uso da Krigagem indicativa (estimação geoestatística), é possível a construção de uma superfície espacial de probabilidades de ocorrências, que pode ser uma ferramenta útil para implementação de políticas públicas. A análise multicritério no ambiente do Sistema de Informação Geográfica permitiu a compreensão dos fatores espaciais relacionados ao fenômeno em análise.

## INTRODUCTION

Daily, in the urban road space of major Brazilian cities, drivers, fare collectors, and passengers are exposed to noise and atmospheric pollution, traffic congestion and crashes, accidents at work, and acts of violence.

Violence in public transportation affects one of the citizen's rights – mobility –, in addition to being effectively related to public health. The danger inside buses (occurrence of accidents during the journey and acts of violence) compromises the quality of transport in cities^6^.

Quality in public transportation began to be understood, from the 1990s, in the context of quality parameters in the view of users; that is, it was no longer analyzed in the operational field, and it went on to contemplate the expectations and needs of citizens in relation to public transportation. Thus, quality is a quantitative parameter, as well as a qualitative one, since it involves the satisfaction of the user^4^.

The reduction in the quality of the public transportation service causes greater demand for the use of cars and other individual travel modes. The consequences of this problem are easily perceived by the increase of vehicles in roads, increasing the occurrence of traffic congestion and car crashes and, consequently, increasing the time in the traffic. Congestion is seen as one of the main factors that affects the quality of life of persons and the efficiency of the transportation system^3^.

In this way, public transportation should be valued over the individual motorized transport, providing quality and efficiency, according to the mobility needs of the population. This involves an improvement in safety related to the public transportation system, especially reducing robberies in buses.

In the approach of violence in urban buses, the problem of robbery corresponds to the dimension of public safety, together with other problems, such as theft, harassment, and social and political violence. Studies show that the decrease in the number of bus users, especially in the less busy hours, is due to the high incidence of robberies^13^.

Mendes^14^ has analyzed robberies in buses in the city of Uberlândia, state of Minas Gerais, Brazil, between 2005 and 2006, and has identified some characteristics of the neighborhoods that had the highest number of robberies, namely high passenger demand and peripheral location. Specifically in Salvador, state of Bahia, Brazil, Paes-Machado and Levenstein^16^ have developed a study that addresses the impact of violent crime on working conditions, health, and safety of public transportation workers, using a qualitative method. With the analysis of the profile of the perpetrators involved in crime in urban buses, the authors have observed that they are generally young, unemployed, and without criminal records.

Assunção and Medeiros^2^ have tested whether sociodemographic factors and working conditions were associated with violence against bus workers in a metropolitan area (three cities in the Belo Horizonte Metropolitan Area, Brazil). In this study, the age of the road worker was inversely associated with violence. Chronic diseases, absenteeism-disease, and working conditions were also associated with violence^2^.

A study developed by Hart and Miethe^8^ has analyzed the violence around bus stops and at other system nodes in Australia. The authors have based their study on the elements of environmental criminology for the analysis and they have identified that bus stops are the most likely locations of robbery when compared to any other node in the network.

Some cities have more frequent cases of violence in public transportation in certain places, and spatial analysis can be used for studies on the phenomenon. Taking into account the literature review, robberies in buses have a territorial dimension and, generally, a spatial distribution pattern, which allows the insertion of techniques that consider the spatial attribute, such as the use of geostatistics. Thus, the proposal of this study is to incorporate spatial techniques, enhancing the analyses using an additional attribute, the geographical location of the events. From geostatistical techniques, we can identify the spatial structure of the data and know aspects that would not be available by traditional methods. Considering the mentioned technique, we can estimate the probability of occurrence of robberies in collective travel mode in geographic coordinates of known and unknown values of the variable of interest.

The second spatial tool used in this study to investigate spatial factors related to robberies in buses is the multicriteria analysis in Geographic Information System (GIS). It is a technique that allows us to evaluate and add many criteria, which are later represented in the analysis as geographic data plans^6^. Thus, this study aims to perform a spatial analysis of the occurrence of robberies in public transportation using geostatistical approach (indicator Kriging), as well as multicriteria analysis in GIS.

## METHODS

The main materials used in this research were auxiliary software and data. The data used on robberies in urban buses refer to the occurrences recorded in 2013 by the Statistical Management System (SGE), of the Department of Public Security of Bahia (SSP-BA)^17^. These data were provided by the Superintendency of Integrated Management of Police Action (SIAP), from the same agency.

**Figure 1:**
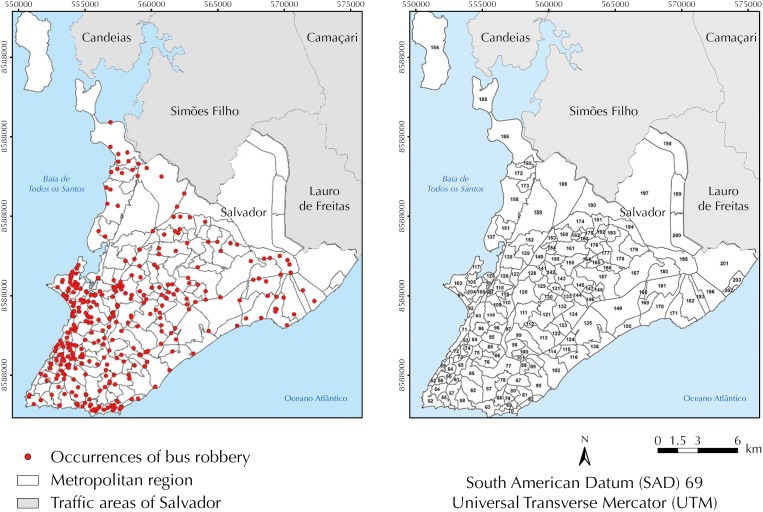
Distribution of the centroids of the streets with record of robbery in buses (A) and traffic analysis zones (B). Salvador, state of Bahia, Brazil, 2013.

Sources: Department of Public Security of Bahia^17^ (2013). Department of Infrastructure of Bahia^18^ (2012).

The SGE has a georeferenced database of the streets of Salvador. Therefore, all records in this system have coordinates of the centroids of the street where the crime happened. Specifically in relation to the crime of robbery in buses in 2013, of the total of 1,210 records made in the period, 969 have coordinates (Figure 1, A). As the information on the coordinate of the crime was essential to quantify the robberies by traffic analysis zone (TAZ), only 969 records were considered.

The unit of analysis of this study corresponds to 151 TAZ of Salvador (Figure 1, B). These areas, published in the last survey named *Origem-Destino* ^18^, had their limits defined by making them compatible with the limits of municipalities and census tracts of IBGE (2010)^9^.

The steps that contemplate the geostatistical analysis of this study were carried out in the program ArcGis (Environmental Systems Research Institute – ESRI), as well as the total robberies by TAZ and the maps resulting from indicator Kriging. The multicriteria evaluation was performed using the software IDRISI.

This research followed two major steps: (1) geographic investigation of the phenomenon – Geostatistical Modeling, and (2) analysis of possible causes: multicriteria analysis in GIS.

The first major step corresponds to geostatistical modeling. Geostatistics emerged from the second half of the twentieth century. Later, the French engineer Matheron (1963)^12^, analyzed the principles of this technique and proposed the concept of regionalized variables, and he also introduced the concept of variogram.

Since then, several studies have emerged with the application of geostatistical techniques, mainly in the geology^5^ ^,^ ^11^ ^,^ ^19^ and hydrology^20^ areas, as well as in oil and gas studies^4^. More recently, we can observe the use of this technique in the area of public health, which allows the spatial estimation of the occurrence of diseases, homicides, and urban violence^10^ ^,^ ^15^ ^,^ ^20^.

The regionalized variable refers to the fact that the observed values are not totally independent, since they have influence from their geographical location^1^. Therefore, an area with a high value sample is more likely to have similar values in its proximity.

In this paper, the regionalized variable was the number of robberies in buses, associated with the geographic coordinates of the centroids of the traffic analysis zone. This variable was transformed into indicator (0 or 1), taking into account the chosen cutoff value, to apply the indicator Kriging.

Thus, the geostatistical estimation of robberies in urban buses was performed by transforming a counting variable (amount of robberies) into a dichotomous variable (zero < cutoff value, 1 ≥ cutoff value). The cutoff value chosen corresponds to the value of the 3rd quartile.

The geostatistical modeling starts by obtaining the experimental variograms and adjusting the theoretical variograms. The variogram graphically represents the regionalized variable information. The function of the variogram is determined by the mean of the variances between the points, as in the following equation:

$$\gamma \left(h\right)=\frac{1}{2N}\sum_{i=1}^{n\left(h\right)}{\left[Z\left(x\right)-Z\left(x+h\right)\right]}^{2}$$

Where *N* is the total number of observations of the sample in each distance *h*.

From the mathematical concepts established in the equation, the experimental variogram is graphically represented. The next step refers to the adjustment of the theoretical variogram (spherical and exponential models, for example). The parameters of the adjusted variograms (nugget effect, sill, and range) are used in the estimation step (Kriging) as weights for interpolation.

**Figure 2:**
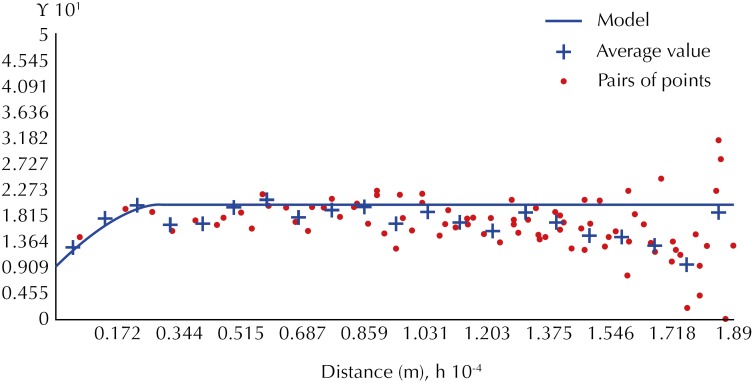
Adjusted Variogram for the binary variable bus robbery: 3rd Quartile (Parameters of the model: Type: Spherical. Main direction: 37.1°; Nugget Effect: 0.091; Range: 2,600,00 m; Sill: 0.187). Salvador, state of Bahia, Brazil.

Thus, we carried out the adjustment of the theoretical models and obtained important parameters for estimation, such as the nugget effect, sill, and range. Figure 2 shows the result of the theoretical variogram.

To verify the quality of the theoretical variogram model, we need the cross-validation step, which is performed by comparing the real and estimated values of the regionalized variable (RV) under analysis. The observed point is ignored, and a new value is estimated based on the weights of the neighboring samples, set by the theoretical variogram. Finally, for each point of the same coordinate, we have the known value and the estimated value, and we can measure the reliability of the results using statistical measures of performance. In the case of indicator Kriging, the cross-validation result was the probability of occurrence of the phenomenon. After discretizing the estimated probabilities and transforming them into values of 0 and 1, appropriate tests for qualitative variables can be used. In this paper, we analyzed only the percentage of hit rates of the geostatistical estimation.

The step following the cross-validation corresponds to the estimation by indicator Kriging. Kriging allows us to predict values in non-sampled locations as well as provide an estimate of the point with an associated measure of accuracy. One of the objectives of this measure is to minimize errors related to data forecasting. Then, we have a weighting of the samples, which generally reduces the excess errors, which is the main advantage of Kriging^1^.

Indicator Kriging is part of the category of nonlinear Kriging estimators. Its analysis involves from the transformation of the original data, calculation, and modeling of the variogram to the estimation of the probability of occurrence of the analyzed phenomenon^21^. This type of kriging has the advantage of being unaffected by outliers, thus presenting easily adjustable variograms when compared to the variograms of the original data^1^. The interpolated maps obtained by Kriging are presented in the results section.

The second great methodological step corresponds to understanding the possible causes for robberies in public transportation. Thus, we carried out a multicriteria analysis in GIS. In order to carry out the multicriteria evaluation, we considered the following factors identified by the experts as being the ones that most influence the occurrence of robberies in buses: F1 - proximity to places that facilitate escape, F2 - absence of policing, F3 - higher bus frequency, F4 - great movement of persons, and F5 - proximity to points of drug trafficking.

We consulted public security experts who work directly against robberies in buses and persons who have developed research on the subject. Thus, from the application of a priority matrix, experts weighted each factor relative to each other in a peer-to-peer comparison, respecting the intensities of importance – “much more important” (10), “more important” (5), “equally important” (1), “less important” (0.2), “much less important” (0,1) – in order to determine the weights of each factor.

The final scores attributed to each factor were considered to determine their respective weights. Thus, the combination of all factors in the multicriteria evaluation respected the weights from the priority matrix analyzed by the experts.

After that, the information plans that represent each factor were mapped from the information collected in different sources and the pre-processing, when necessary, preliminarily using the vector format for representation. After processing and updating the information used to map each factor, we transformed the file into *raster* and we carried out the normalization and fuzzy classification of the data (ranging from 0 to 1).

Thus, all mapped factors were integrated in the multicriteria analysis by the linear combination method weighted in GIS environment. The product of the weights of the factors by the values of the factors determined the variable called “robbery potential”. The calculation of the “robbery potential”, in this paper, was carried out in three sections belonging to the region determined as critical in the geostatistical modeling.

We highlight that this is a sequential and spatial approach to a specific case of urban violence. The first step – geostatistical modeling – is intended to determine geographically potential areas for the occurrence of robberies in collective travel mode. The final step – multicriteria analysis in GIS – corresponds to the understanding of the factors related to the crime – robbery in buses.

## RESULTS

The presentation of the results is summarized in two main steps: (i) presentation of the map of estimated values, and (ii) presentation of the results of the multicriteria analysis in GIS.

### Presentation of the Maps of Estimated Values

Cross-validation tests the quality of the adjusted or theoretical variogram model for the cutoff value of the variable “number of robberies in buses” equivalent to the 3rd quartile. After verifying the quality of the variographic models (in this case, the accuracy was 75%), the parameters of the theoretical variograms are used in the next step of the geostatistical modeling – indicator Kriging.

**Figure 3:**
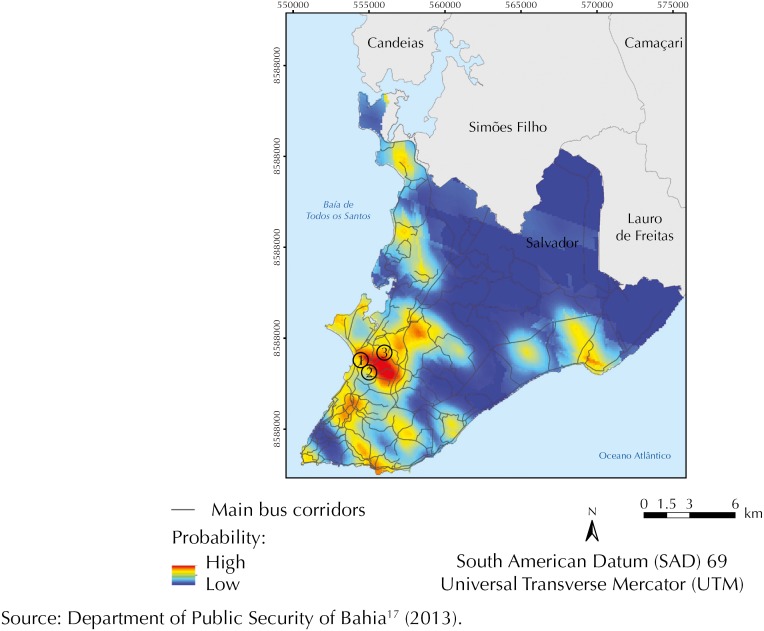
Map of probability of occurrences of robbery in buses. Salvador, state of Bahia, Brazil, 2013.

Source: Department of Public Security of Bahia17 (2013).

Thus, surface maps or maps of estimated values are generated considering a Kriging grid, variable values, spatial locations, and parameters of theoretical variograms. Figure 3 presents the surface map, which estimates the probability of the analyzed variable. Areas with shades closer to red correspond to the greater risk of robberies above the cutoff value, while areas with shades of blue correspond to the lowest probability. We also marked in the same figure the road sections that were part of the multicriteria analysis, located in the region with greater probability of robberies.

### Presentation of the Results of the Multicriteria Analysis in GIS

The final scores attributed by five experts to each factor are listed below and were considered to determine their respective weights. We can note that the factor with the highest weight corresponded to F2 - absence of policing, with 0.32.

- F1 - proximity to places that facilitate escape (weight = 0.26);
- F2 - absence of policing (weight = 0.32);
- F3 - higher frequency of buses (weight = 0.17);
- F4 - great movement of persons (weight = 0.13);
- F5 - proximity to points of drug trafficking (weight = 0.12).

**Figure 4:**
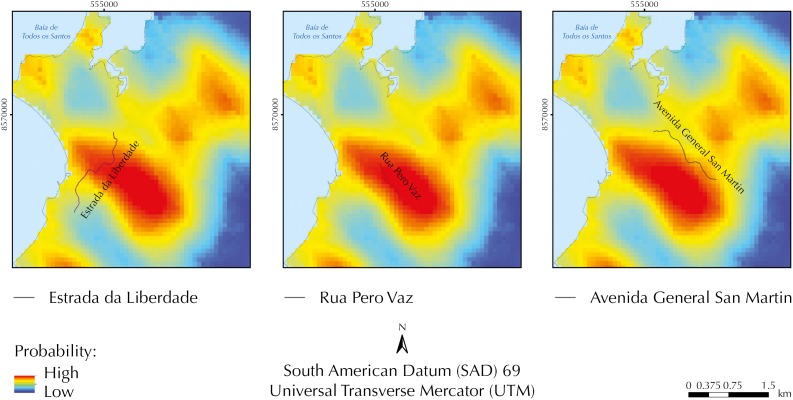
Map of probability of occurrences of robbery in Buses. Salvador, state of Bahia, Brazil, 2013.

After mapping the values corresponding to each factor in the urban space and calculating the product between their respective weights and assigned values, we calculated the variable “robbery potential” in three road sections in the most critical area. The analyzed sections are illustrated in Figure 4: Section 1 - *Estrada da Liberdade*, Section 2 - *Rua Pero Vaz*, and Section 3 - *Avenida General San Martin.*

**Figure 5:**
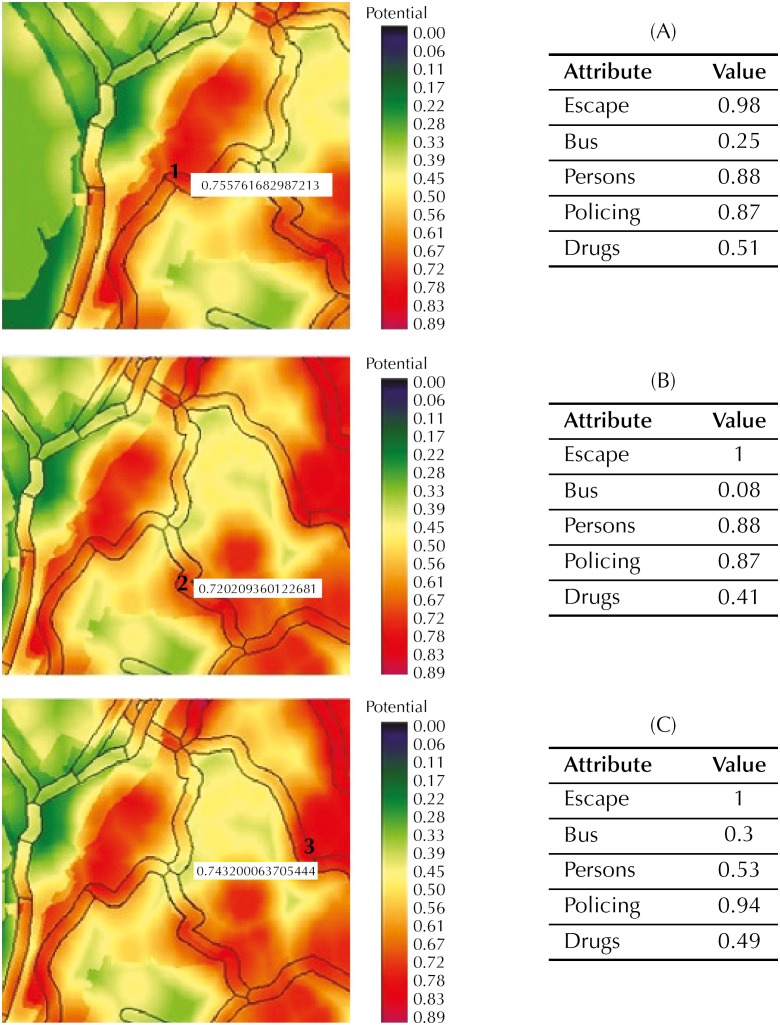
Analysis of the potential for robbery in buses at *Estrada da Liberdade* (Section 1), *Rua Pero Vaz* (Section 2), and *Avenida General San Martin* (Section 3). Salvador, state of Bahia, Brazil.

When analyzing in detail the critical section of the occurrence of robberies in buses at *Estrada da Liberdade* (Section 1), we can observe that the factors that most contribute to this potential are: F1 - proximity to places that facilitate escape, F3 - great movement of persons, and F2 - absence of policing, respectively (Figure 5, A).

Specifically at *Rua Pero Vaz* (Section2), the factor of proximity to sites that facilitate escape (F1) presented maximum contribution to determine the potential for occurrence of robberies in buses, followed by factors F3 - large movement of persons - and F2 - absence of policing -, respectively (Figure 5, B).

At *Avenida General San Martin* (Section 3), factor F1 - proximity to places that facilitate escape - also presents maximum contribution to the potential for robber in buses, followed by factor F2 - absence of policing. The factors that had the smallest contribution were F3 - higher bus frequency - and F5 - proximity to points of drug trafficking, as detailed in Figure 5 (C).

## DISCUSSION

The spatial analysis using indicator Kriging was considered effective in determining the traffic analysis zones and regions of greater probability of robberies in buses. The areas with the highest occurrence of robberies are detected in the analyses obtained by indicator Kriging, such as the central region, peninsula, and center of Salvador.

An advantage of the use of indicator Kriging in relation to other types of Kriging in the prediction of variables with little variability and the presence of outliers is that we can obtain easily adjusted variograms, which extends the predictive power from the spatial relation of the analyzed variable. We can also observe that, with this technique, we can obtain data in points that were not sampled, from a continuous surface of data that shows areas prone to the occurrence of violence in public transportation. In this way, using the methodological procedures mentioned, urban spots are obtained that indicate a greater probability of robberies in buses. From the cutoff value determined in this study, we could identify spots associated with probability of occurrences higher than the value of the 3rd quartile for 2013.

Using geostatistical modeling, considering only the spatial autocorrelation of the regionalized variable, we can estimate such probabilities even in locations of unknown values. The variances of the variable under study in relation to distances (variography) can detect areas of risk in the region of study with good accuracy (75% of hit rates).

A complementary or sequential analysis was the multicriteria analysis in GIS, which we performed in this paper to detect, considering spatial factors, the main causes in the region of greater robbery potential. We observed that the factors that contributed the most to determine the high potential were F1 - proximity to places that facilitate escape, F3 - great movement of persons, and F2 - absence of policing.

The observation of these spatial trends is confirmed by geostatistics. Further analysis will allow better investigation of the phenomenon. Thus, the use of this methodological procedure can aid in the process of urban and regional planning and in the implementation of public security policies aimed at ensuring the right to mobility.
